# Physiological and Race Pace Characteristics of Medium and Low-Level Athens Marathon Runners

**DOI:** 10.3390/sports8090116

**Published:** 2020-08-21

**Authors:** Aristides Myrkos, Ilias Smilios, Eleni Maria Kokkinou, Evangelos Rousopoulos, Helen Douda

**Affiliations:** 1 School of Physical Education and Sport Science, Democritus University of Thrace, 69100 Komotini, Greece; aris7tefaa@gmail.com (A.M.); ekokkino@phyed.duth.gr (E.M.K.); edouda@phyed.duth.gr (H.D.); 2 Ergoscan Physical Performance Evaluation Center, Ionias 110, 17456 Alimos, Greece; vrousso@gmail.com

**Keywords:** endurance, aerobic performance, lactate threshold, running economy, maximal oxygen consumption, oxygen fractional utilization, running

## Abstract

This study examined physiological and race pace characteristics of medium- (finish time < 240 min) and low-level (finish time > 240 min) recreational runners who participated in a challenging marathon route with rolling hills, the Athens Authentic Marathon. Fifteen athletes (age: 42 ± 7 years) performed an incremental test, three to nine days before the 2018 Athens Marathon, to determine maximal oxygen uptake (VO_2_ max), maximal aerobic velocity (MAV), energy cost of running (ECr) and lactate threshold velocity (vLTh), and were analyzed for their pacing during the race. Moderate- (*n* = 8) compared with low-level (*n* = 7) runners had higher (*p* < 0.05) VO_2_ max (55.6 ± 3.6 vs. 48.9 ± 4.8 mL·kg^−1^·min^−1^), MAV (16.5 ± 0.7 vs. 14.4 ± 1.2 km·h^−1^) and vLTh (11.6 ± 0.8 vs. 9.2 ± 0.7 km·h^−1^) and lower ECr at 10 km/h (1.137 ± 0.096 vs. 1.232 ± 0.068 kcal·kg^−1^·km^−1^). Medium-level runners ran the marathon at a higher percentage of vLTh (105.1 ± 4.7 vs. 93.8 ± 6.2%) and VO_2_ max (79.7 ± 7.7 vs. 68.8 ± 5.7%). Low-level runners ran at a lower percentage (*p* < 0.05) of their vLTh in the 21.1–30 km (total ascent/decent: 122 m/5 m) and the 30–42.195 km (total ascent/decent: 32 m/155 m) splits. Moderate-level runners are less affected in their pacing than low-level runners during a marathon route with rolling hills. This could be due to superior physiological characteristics such as VO_2_ max, ECr, vLTh and fractional utilization of VO_2_ max. A marathon race pace strategy should be selected individually according to each athlete’s level.

## 1. Introduction

Marathon running is one of the most demanding races which requires well-organized mental and physical preparation [1]. Today, marathon races have turned into very large events where thousands of elite, high-level and recreational athletes participate in this 42.195 m race [2,3,4]. For many years, the physiological demands of a marathon as well as the physiological characteristics of top-class athletes were examined by researchers [5,6,7,8,9,10,11]. It is known that the most important parameters to sustain the highest possible running velocity over a marathon are the maximal oxygen uptake (VO_2_ max), a high fractional utilization of VO_2_ max and the energy cost of running (ECr) [7,12,13]. These parameters explain 70% of the variance of the average running speed sustained during a marathon race [6,7] and are good indicators of the endurance performance of individuals of different ages, genders and disciplines [1]. A typical VO_2_ max value for male top-class marathoners is about 70–85 mL/kg/min, for low-level athletes around 65 mL/kg/min and for recreational runners about 51–58 mL/kg/min [14,15,16]. Additionally, oxygen fractional utilization at lactate threshold (LTh) intensity, the point where blood lactate concentrations increase from baseline, is higher for top-class marathoners compared with low-level athletes (65–80% vs. 50–80% of VO_2_ max, respectively) and is also higher at the lactate turn-point (LTP), the point where an abrupt increase in blood lactate is observed (85–90% vs. 80–85% of VO_2_ max, respectively) [6,11,12,17,18].

Few studies have examined in more detail the physiological characteristics of recreational marathon runners, with finishing times >3 h, and how these characteristics affect performance in this group of runners. It was shown that the better the level of recreational marathoners, the higher the VO_2_ max as well as the velocity and the VO_2_ at LTh [2,19]. No differences were observed between the different level of runners in the LTh expressed as a percentage of VO_2_ max and the oxygen cost of running at LTh [2]. Regarding medium- and low-level recreational runners, however, no data exist about the correspondence of race pace on the blood lactate curve, the fractional utilization of VO_2_ max at race pace and if these differ according to the performance ability of the runners.

Most of the studies examined runners who participated in a marathon ran on a flat terrain where they could sustain a relatively stable pace till the end of the race, although the lower the level of the runners, the higher the variability in race pace [20]. The peculiarity of the terrain could be an external factor that may affect the physiological and race pace characteristics of a marathon race. The terrain at one of the most famous and challenging marathons in the world, the Athens Authentic Marathon, is characterized by rolling hills and includes the toughest uphill climb of any major marathon. The total ascent is 317 m (51.2% of the route is uphill), the total descent is 262 m (40.5% of the route is downhill) and the steepest grading ranges from −6.2 to 3.8% [21,22]. It is possible that the difficulty of the route may affect differently the race pace characteristics of medium- and low-level recreational runners. A runner with a faster pace will cross the hill segments in a shorter amount of time compared with a slower runner, altering probably the physiological requirements of the run. Therefore, recreational runners of different levels may run the Athens Marathon at a rate corresponding to different percentages of aerobic performance parameters. This may lead athletes and coaches to over- or underestimate the potential performance and to the determination of a false race pace strategy. Therefore, it would be useful to examine which are the physiological and race pace characteristics of medium- and low-level recreational athletes participating in the Athens Marathon and if they adopt different pace characteristics in relation to their physiological profile. Based on the above, the aim of the present study was to compare the physiological and race pace characteristics of medium- (finish time < 240 min) and low-level (finish time > 240 min) recreational runners who participated in the Athens Marathon.

## 2. Materials and Methods

### 2.1. Participants

Fifteen recreational marathon runners (age: 42 ± 7 years, height: 174.9 ± 6.5 cm and body mass: 72.8 ± 6.9 kg) volunteered to take part in the study. All participants were healthy and ran approximately 1–2 years on a systematic basis with a structured program with an average weekly load of 50–60 km. Based on their finishing time at the Athens Marathon 2018, athletes were divided into a moderate-level group, with finishing times < 240 min (*n* = 8), and a low-level group with finishing times > 240 min (*n* = 7). Before the start of the study, the institutional review board committee approved the experimental protocol in accordance with the Helsinki Declaration.

### 2.2. Maximal Incremental Test

Three to nine days before participation in the Athens Marathon 2018 race, participants performed a maximal incremental test on a treadmill (Technogym run race 1200, Italy) for the determination of VO_2_ max, maximum aerobic velocity (MAV), maximum heart rate (HRmax), the relationship between blood lactate concentration and running velocity, oxygen consumption and running velocity, heart rate and running velocity, and the energy cost of running.

The protocol started at 7 km·h^−1^ and was increased by 1.5 km·h^−1^ every 3 min until volitional exhaustion. Treadmill grade was set at 1% throughout the protocol. Gas exchange was measured by the open circuit Douglas bag method as described by Cooke (2009). The subject breathed through a low-resistance 2-way Hans-Rudolph 2700 B valve (Shawnee, OK, USA). The concentrations of CO_2_ and O_2_ in the expired air were measured by using the Hi-tech (GIR 250) combined Oxygen and Carbon Dioxide Analyzer. The gas analyzers were calibrated continuously against standardized gases (15.35% O_2_, 5.08% CO_2_ and 100% N_2_). Expired volume was measured by means of a dry gas meter (Harvard) previously calibrated against standard air flow with a 3 L syringe. Barometric pressure and gas temperature were recorded and respiratory gas exchange data for each work load (i.e., VO_2_, VCO_2_ and V_E_) were determined based on the computations described by Cooke [23] when V_E_atps, FECO_2_ and FEO_2_ are known. The highest VO_2_ value obtained during a 30-sec time period during the incremental exercise test was recorded as the subject’s $\dot{V}O_{2}\max$. HR was continuously measured telemetrically (Polar RS400) and the highest 10 sec value was regarded as maximal. The test was considered as maximal when at least 3 of the following criteria were achieved: (a) visual exhaustion of the participants, (b) a plateau in oxygen consumption (<2 mL kg^−1^·min^−1^) despite an increase in running velocity, (c) maximal HR higher than 90% of the predicted maximum (220-age) and (d) maximum respiratory exchange ratio > 1.1. MAV was calculated using the following formula: MAV (km·h^−1^) = Velocity of the last completed stage + (seconds run at last stage/180).

### 2.3. LTh and LTP Determination

At the end of each stage during the incremental test, approximately 0.3 μL of whole blood was collected from the fingertip and immediately analyzed for lactate concentration with a portable analyzer (Lactate Pro 2, Arkray Factory Inc., Koka-Shi, Japan) using an enzymatic-amperometric method. The individual relationships between blood lactate concentrations and running velocities were determined using an exponential model: y = a + b × exp(x/c), where y = lactate concentration, x = running velocity and a, b and c are constants. The LTh and the LTP were identified as the velocities (km·h^−1^) at which blood lactate concentrations were increased by 0.3 and 1.5 mmol·L^−1^ from baseline values, respectively. Furthermore, LTh and LTP were expressed relative to MAV (%MAV) units, and based on the relationship between VO_2_ and running velocity were also expressed in absolute (mL·kg^−1^·min^−1^) and relative (%VO_2_ max) VO_2_ max values.

### 2.4. Energy Cost of Running

The gas exchange data (VO_2_, VCO_2_) collected during the final 30 s of every 3-min stage up to the previous stage from the LTP were used for the calculations of the caloric cost of running. Substrate oxidation rate (g·min^−1^) was estimated using nonprotein respiratory quotient equations [24]:

Fat oxidation (g·min^−1^) = 1.6947 × VO_2_ (L·min^−1^) − 1.7012 × VCO_2_ (L·min^−1^)

Carbohydrate oxidation (g·min^−1^) = 4.5851 × VCO_2_ (L·min^−1^) − 3.22259 × VO_2_ (L·min^−1^)

The energy produced from each substrate was calculated by assuming an energy equivalent for 1 g of fat and carbohydrate of 9.75 and 4.07 kcal, respectively [25]. Total ECr was quantified from the sum of these values and was expressed in kcal·kg^−1^·km^−1^. The energy cost of running at 10 km/h and at the velocities corresponding to LTh and marathon race pace were estimated from the relationship between exergy cost and running velocity derived from the incremental test.

All the physiological data were analyzed after the completion of the marathon race to avoid any pacing strategies from the participants and their coaches based on the results of testing.

### 2.5. Route Characteristics and Race Pace Analysis

The profile of the Athens Marathon route includes rolling uphills and downhills. More specifically, when calculated in 450 m intervals, the total ascent, total descent, the percent of uphill distance, the percent of downhill distance and the steepest uphill and downhill, respectively, are for: (a) the total route: 317 m, 262 m, 51.2%, 40.5%, 3.8% and −6.2%, (b) the 0–10 km split: 19 m, 36 m, 36.4%, 50%, 1.3% and −2.0%, (c) the 10–21.1 km split: 143 m, 66 m, 66.7%, 25%, 3.6% and −6.2%, (d) the 21.1–30 km split: 122 m, 5 m, 95%, 5%, 3.3% and −1.1% and (e) the 30–42.195 km split: 32 m, 155 m, 15.4%, 76.9%, 3.8% and −5.1% [22].

Finishing time and split times for each participant were exported from the official results posted on the site of the organization [21]. The average running velocity of each runner was calculated by dividing marathon distance to the time needed to complete the race. Race pace was expressed as a percentage of VO_2_ max (index of fractional utilization of VO_2_ max), MAV and the velocities at LTh (vLTh) and LTP (vLTP).

To determine the differences between the two groups in pacing during the race, average running velocities for the distances of 0–10, 10–21.1, 21.1–30 and 30–42.195 km were calculated by dividing the distance of the split to the time to complete the split. For the analysis of the data, mean velocity of each split was expressed as a percentage of the vLTh. The velocity at LTh was selected as a reference point because for the whole sample, average marathon running velocity was equal to vLTh.

### 2.6. Statistical Analysis

All data are presented as means ± SD. Normality of the distribution of the data was examined with the Shapiro–Wilk’s W test. A *t*-test was used to examine the differences among the medium-level and the low-level runners in the physiological parameters and race pace characteristics measured. A two-way analysis of variance with repeated measures in the second factor was used to examine the differences between the two groups in the mean velocity of each running split (0–10, 10–21.1, 21.1–30 and 30–42.195 km). Significant differences between means were located with the Newman–Keuls post hoc test. Pearson product moment correlations were used to determine the association between marathon time and the measured parameters. The statistical significance level was set for all tests at *p* < 0.05.

## 3. Results

### 3.1. Physiological Characteristics

Medium-level runners had higher (*p* < 0.05) VO_2_ max, MAV, LTh (km·h^−1^), LTh (%MAV), LTh (mL·kg^−1^·min^−1^), LTP (km·h^−1^), LTP (%MAV) and LTP (mL·kg^−1^·min^−1^) than the low-level group. There were no significant differences (*p* > 0.05) between groups at HR_max_, LTh (%VO_2_ max) and LTP (%VO_2_ max) (Table 1). Medium-level runners had lower ECr at 10 km·h^−1^ (*p* = 0.05), at vLTh (*p* = 0.07) and at marathon race pace (*p* = 0.09) (Table 1).

### 3.2. Race Pace Characteristics

**Figure 1:**
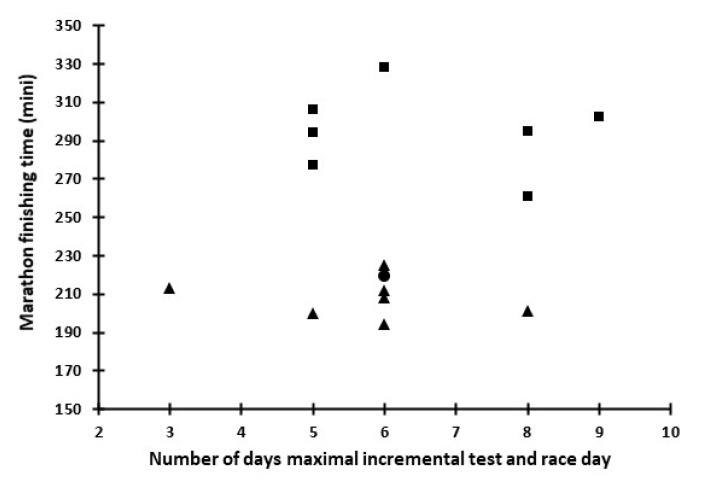
Plot of marathon finishing time vs. number of days between maximal incremental test and race day for the low-(squares) and the medium-level runners (triangles).

**Figure 2:**
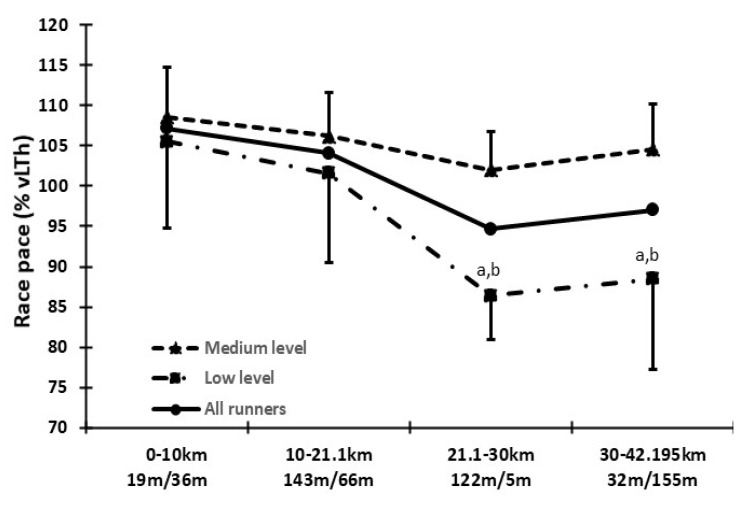
Race pace, expressed as a percentage of the velocity at lactate threshold (%vLTh), at the running splits of 0–10, 10–21.1, 21.1–30 and 30–42.195 km (total ascent in meters/total decent in meters) of the Athens Marathon, for the low-level, the medium-level and all runners. a: *p* < 0.05 significant difference between low- and medium-level runners, b: *p* < 0.05 significantly different from the 0–10 and 10–21.1 km splits for the low-level runners.

Medium-level runners had, by design, a lower (*p* < 0.05) marathon time (209.0 ± 10.4 min, range: 194–225 min) than the low-level runners (289.7 ± 25.1 min, range: 260–328 min). Marathon finishing time was not related to the number of days between the maximal incremental test and the race day (Figure 1). Medium-level runners had a higher (*p* < 0.05) race pace expressed as %MAV, %vLTh, %vLTP, %VO_2_ max and %HRmax (Table 1). Medium- and low-level runners had a similar (*p* > 0.05) race pace (expressed as %vLTh) at the first two running splits (0–10 and 10–21.1 km). However, low-level runners had a lower (*p* < 0.05) race pace at the last two splits (21.1–30 and 30–42.195 km) compared to the medium-level runners (Figure 2).

### 3.3. Correlation between Marathon Time and Measured Variables

Marathon finish time correlated significantly (*p* < 0.05) with VO_2_ max (r = −0,76), MAV (r = −0.88), vLTh (km·h^−1^; r = −0.91), vLTP (km·h^−1^; r = −0.88), LTh (%MAV; r = −0.58), LTh (mL·kg^−1^·min^−1^; r = −0.86), LTP (mL·kg^−1^·min^−1^; r = −0.80), ECr 10 km·h^−1^ (r = 0.62), ECr vLTh (r = 0.59), ECr race pace (r = 0.55), race pace (%VO_2_ max; r = −0.62), race pace (%vLTh; r = −0.75), race pace (%vLTP; r = −0.81) and race pace (%MAV; r = −0.90). Marathon finish time did not correlate significantly (*p* > 0.05) with LTP (%MAV; r = −0.38), LTh (%VO_2_ max; r = −0.22) and LTP (%VO_2_ max; r = −0.21).

## 4. Discussion

The purpose of this study was to provide further insight into the physiological and race pace characteristics of medium- and low-level marathon runners with a completion time < 240 min and > 240 min, respectively, of the Athens Authentic Marathon. This marathon race is famous, not only for historical reasons but also for its level of difficulty due to the peculiarity of the terrain. The results of the present study show that recreational medium-level runners compared to lower-level runners have: (a) higher VO_2_ max, MAV and lactate threshold values in absolute velocity (km·h^−1^) and VO_2_ (mL·kg^−1^·min^−1^) units, (b) higher lactate threshold in relative velocity units (%MAV), (c) lower energy cost of running at 10 km/h and (d) adopt a race pace corresponding to a higher percentage of their lactate threshold velocity and fractional utilization of VO_2_ max and show no significant alterations in their pace due to terrain alterations in contrast to the low-level runners to whom the uphill part of the race leads to great reductions in race pace.

Previous studies have examined the importance of physiological parameters and race pace characteristics of elite marathoners, but few studies provide data for recreational runners [2,19,20]. Maximal oxygen consumption, a high fractional utilization of VO_2_ max and the energy cost of running are considered the determinants of endurance performance [26]. Indeed, in the present study the medium-level runners had higher VO_2_ max than the low-level runners. This agrees with previous reports where the better level marathoners had higher VO_2_ max than the lower level [2,7,18,19]. The VO_2_ max values of the medium-level marathoners (55.56 ± 3.62 mL/kg/min) measured in the present study are approximately the same (55.7 ± 4.8) as those reported by Gordon et al. [2] for athletes who ran the marathon between 3:00 and 3:30 h as in the present study. The same holds even for the low-level runners (VO_2_ max: 48.85 ± 4.77 mL/kg/min; finish time: 4:00–5:30 h) of the present study and runners with approximately similar finishing times in Gordon et al.’s [2] (VO_2_ max: 46.5 ± 5.2 mL/kg/min; finish time: >4:30 h) and Chmura et al.’s [14] (VO_2_ max: 51 ± 2 mL/kg/min; finish time: 4:17 ± 10.51 min) studies. It appears that certain levels of VO_2_ max are necessary to achieve certain marathon times regardless of the level of the runner since VO_2_ max determines the upper limit of aerobic performance. The high correlation between VO_2_ max and marathon performance which has been previously reported for high-level to elite athletes [7,18,27,28] supports this notion. A large correlation (r = −0.76) between VO_2_ max and marathon performance was observed as well in the present study for medium- to low-level marathon runners, enriching the limited information available for recreational athletes [2,19].

For most sports scientists, running economy or energy cost of running is a key factor for performance in long distance events and becomes more important as running distance increases [29,30,31,32]). In the present study, we examined ECr at a specific speed (10 km/h) and at the vLTh and we found that medium-level runners had lower ECr than the lower-level runners. This was probably another factor that allowed them to run the marathon at a faster pace. It should be noted that the ECr in the medium-level runners tended to be lower in the race pace as well. This is of importance considering that the medium-level runners sustained a faster running pace. Furthermore, ECr at 10 km/h and at vLTh had large correlations with marathon time (r = 0.62 and r = 0.59, respectively). The results of the current study reveal that running economy is a determinant of performance even for recreational runners with limited training experience and supports the suggestion in the literature that athletes should focus their training on the optimization of this parameter as well [32,33,34].

Besides the importance of VO_2_ max and energy cost of running for marathon performance, stronger associations are observed between maximal aerobic velocity and the velocities at the lactate threshold or any point on the blood lactate curve, and endurance performance [35,36,37]. Similarly, very large correlations were observed in this study between marathon time and velocities at LTh and LTP (r = −0.91 and −0.88) and MAV (r = −0.88) of recreational runners. The velocity at LTh was the stronger single predictor of marathon finish time. This is not surprising considering that these indexes, when expressed in velocity units, encompass both VO_2_ max and running economy [37]. When LTh and LTP values were normalized to MAV and VO_2_ max, the relationship of these parameters with running performance became lower (r = −0.22 to −0.58). This is because the effect of VO_2_ max and/or running economy was diminished [37]. Medium-level runners had higher LTh values, expressed either in velocity or VO_2_ units, than the lower-level runners. Even when LTh velocity was normalized to MAV, medium-level runners had a higher LTh, indicating a higher ability of the fat oxidation rate to meet ATP demands and the occurrence of a later increased stimulation of glycolysis and glycogenolysis relative to their maximum performance. This probably reflects a greater aerobic capacity and increased buffering capacity promoting the ability to achieve higher running velocities due to metabolic and/or locomotor reasons.

Many studies declare that the fractional utilization of VO_2_ max at LTh, LTP and at race pace is one of the most crucial parameters of aerobic performance along with VO_2_ max and running economy [6,7,31]. Fractional utilization of VO_2_ max at LTh and LTP did not differ between the two groups in the present study. Similarly, Gordon et al. [2] did not find any differences in fractional utilization of VO_2_ max at LTh and LTP between recreational runners with different marathon finish times. It could be that adaptations in the utilization of oxygen from working muscles may require a significant amount of training load which was not achieved by our runners. In the present study, however, we found that medium-level runners had a higher fractional utilization of VO_2_ max at marathon race pace. This agrees with previous findings that in high-level athletes, increased levels of fractional utilization of VO_2_ max at marathon race pace were associated with faster performance [1,2,7]. In addition, a positive correlation of fractional utilization at race pace and marathon time was found (r = −0.62), Therefore, our data reveal that even in recreational runners, fractional utilization of VO_2_ max at marathon race pace appears to be a contributing factor to performance.

A main finding of the present study is that medium-level marathoners ran the marathon distance with an average speed corresponding to higher percentages of vLTh and MAV. The better running economy may allow them to adopt a higher running velocity. Furthermore, the higher LTh and LTP velocities mean that the medium-level runners will cover a given distance at a shorter time. This may allow them to run at a higher point on the blood lactate curve because they can sustain this pace for the time needed to complete the race. On the contrary, the slower LTh and LTP velocities of the low-level runners mean that they need to run for a longer time to complete the race having a lower fractional oxygen utilization. It has been shown that as the duration of an endurance event increases, fractional oxygen utilization decreases [38,39]. The lower running velocity of the low-level runners made them spend more time running the uphill part of the Athens Marathon course. The total ascent from the 21.1st km to the 30th km is about 122 m and almost all this split is uphill. This forced the low-level runners to adopt an even lower velocity during this part of the route. Indeed, the split analysis revealed that the low-level runners were more influenced by this uphill part than the medium level. It appears that this specific segment has the greatest impact in the finish time between different levels of athletes and makes the Athens Marathon a totally different terrain from other marathons. It is worth noting that even at the last part of the route, which is mostly downhill, low-level runners were not able to increase their speed. Probably, the accumulated fatigue after hours of running may increase even more the stress placed on the musculoskeletal system, besides that induced by the increased eccentric load during downhill running, which prevents an increase in running speed compared to the previous uphill part. Therefore, the peculiarity of the terrain may affect differently the performance of a marathon runner depending on his/her ability level. This is of importance for coaches and athletes for the determination of the pace strategy to follow when running on a rolling hill terrain.

An advantage of the present study is that all recreational runners participated in the same marathon race and not in different ones. This makes comparisons between different levels of runners more reliable since all of them competed in the same route, on the same day and under the same environmental conditions. In addition, physiological testing was performed at a time point very close to the race day (three–nine days before) providing valid data about the relationship of physiological determinants of endurance performance and actual marathon running performance. Limitations of the present study, though, should also be acknowledged. A larger sample size would have given more valid data about the different levels of marathon runners. It was difficult, however, to measure many runners at a time close to the actual race. Furthermore, the energy cost of running at 10 km/h and at the velocity corresponding to LTh was estimated from the relationship between exergy cost and running velocity derived from the incremental test. Measurements at the exact velocities would have given more precise values of the energy cost. Again, the execution of these submaximal measurements would have increased the time of testing and it would not be possible to perform them near the race date.

## 5. Conclusions

The results of the present study enrich the existing literature regarding the physiological profile and the race pace characteristics of recreational marathon runners competing in a difficult route, the Athens Marathon. Medium-level runners (finish time range: 194–225 min) have higher VO_2_ max, lactate threshold values, better running economy, greater oxygen fractional utilization at race pace and adopt a faster race pace in relation to their lactate threshold velocity than low-level runners (finish time range: 260–328 min). Furthermore, medium-level runners show no significant alterations in their pace due to terrain alterations in contrast to the low-level runners to whom the uphill part of the race leads to great reductions in race pace. Therefore, slower runners are more influenced by a hilly terrain and they decrease more their running velocity to complete this part of the race. Thus, careful planning of race pace should be considered so that pacing of the parts before the uphill would be of such an intensity to avoid a large decrease in running velocity at uphill. Therefore, besides the focus on training for the improvement of important physiological parameters related to endurance performance, it is recommended that the selected race pace strategy be applied individually according to each athlete’s level.

## Figures and Tables

**Table 1:** Physiological and race pace characteristics of the medium-level, the low-level and of all runners.

	Medium-Level Runners	Low-Level Runners	All Runners	*p* Value between Groups
Age (years)	41.00 ± 7.69	42.14 ± 7.20	41.53 ± 7.22	0.36
Body height (m)	175.00 ± 6.44	174.71 ± 7.06	174.87 ± 6.49	0.94
Body mass (kg)	72.50 ± 6.58	73.17 ± 7.91	72.81 ± 6.97	0.86
VO_2_ max (mL·kg^−1^·min^−1^)	55.56 ± 3.62	48.85 ± 4.77	52.43 ± 5.32	0.01
MAV (km·h^−1^)	16.45 ± 0.74	14.39 ± 1.24	15.49 ± 1.44	0.01
HRmax (b·min^−1^)	178.25 ± 9.54	183.71 ± 9.25	180.80 ± 9.5	0.28
vLTh (km·h^−1^)	11.58 ± 0.81	9.22 ± 0.72	10.48 ± 1.42	0.01
vLTP (km·h^−1^)	13.6 ± 0.87	11.1 ± 0.8	12.43 ± 1.52	0.01
vLT1% (%MAV)	70.37 ± 3.9	64.25 ± 4.15	67.51 ± 5.00	0.01
vLT2% (%MAV)	82.7 ± 4.83	77.37 ± 4.52	80.21 ± 5.29	0.05
VO_2_ LTh (mL·kg^−1^·min^−1^)	41.76 ± 1.81	35.11 ± 2.80	38.65 ± 4.10	0.01
VO_2_ LTP (mL·kg^−1^·min^−1^)	48.04 ± 2.41	40.80 ± 3.78	44.66 ± 4.80	0.01
%VO_2_ LTh (%VO_2_ max)	75.31 ± 3.64	72.14 ± 5.87	73.83 ± 4.91	0.22
%VO_2_ LTP (%VO_2_ max)	86.59 ± 3.90	83.63 ± 4.08	85.21 ± 4.13	0.17
ECr 10 km·h^−1^ (kcal·kg^−1^·km^−1^)	1.137 ± 0.096	1.232 ± 0.068	1.181 ± 0.096	0.05
ECr vLTh (kcal·kg^−1^·km^−1^)	1.157 ± 0.079	1.232 ± 0.066	1.192 ± 0.081	0.07
ECr Race Pace (kcal·kg^−1^·km^−1^)	1.160 ± 0.083	1.232 ± 0.068	1.194 ± 0.082	0.09
Race pace (km·h^−1^)	12.14 ± 0.60	8.63 ± 0.64	10.50 ± 1.91	0.01
Race Pace (%MAV)	73.82 ± 2.60	60.11 ± 3.13	67.42 ± 7.59	0.01
Race Pace (%vLTh)	105.08 ± 4.71	93.80 ± 6.20	99.82 ± 7.84	0.01
Race Pace (%vLTP)	89.45 ± 4.47	77.92 ± 6.14	84.07 ± 7.85	0.01
Race Pace (%HRmax)	83.91 ± 5.8	77.41 ± 5.40	80.87 ± 6.37	0.04
Race Pace (%VO_2_ max)	79.74 ± 7.65	68.80 ± 5.73	74.63 ± 8.68	0.01

HRmax: maximum heart rate, MAV: maximal aerobic velocity, vLTh: velocity (km·h^−1^) at lactate threshold, vLTP: velocity (km·h^−1^) at lactate turn-point, ECr: energy cost of running.

## References

[1] Zinner C., Sperlich B. (2016). Marathon Running: Physiology, Psychology, Nutrition and Training Aspects.

[2] Gordon D., Wightman S., Basevitch I., Johnstone J., Espejo-Sanchez C., Beckford C., Boal M., Scruton A., Ferrandino M., Merzbach V. (2007). Physiological and training characteristics of recreational marathon runners. Open Access J. Sports Med..

[3] Berndsen J., Smyth B., Lawlor A. Pace my race: Recommendations for marathon running. Proceedings of the 13th ACM Conference on Recommender Systems.

[4] Fokkema T., van Damme A., Fornerod M., de Vos R.J., Bierma-Zeinstra S., van Middelkoop M. Training for a (half-)marathon: Training volume and longest endurance run related to performance and running injuries. Scand. J. Med. Sci. Sports.

[5] Foster C., Daniels J., Yarbrough R. (1977). Physiological and training correlates of marathon running performance. Aust. J. Sports Med..

[6] Di Prampero P.E., Atchou G., Brückner J.-C., Moia C. (1986). The energetics of endurance running. Eur. J. Appl. Physiol. Occup. Physiol..

[7] Billat V., Demarle A., Slawinski J., Paiva M., Koralsztein J.-P. (2001). Physical and training characteristics of top-class marathon runners. Med. Sci. Sports Exerc..

[8] Billat V., Demarle A., Paiva M., Koralsztein J.P. (2002). Effect of Training on the Physiological Factors of Performance in Elite Marathon Runners (Males and Females). Int. J. Sports Med..

[9] Billat V., Petot H., Landrain M., Meilland R., Koralsztein J.P., Mille-Hamard L. (2012). Cardiac Output and Performance during a Marathon Race in Middle-Aged Recreational Runners. Sci. World J..

[10] Schmid W., Knechtle B., Knechtle P., Barandun U., Rüst C.A., Rosemann T., Lepers R. (2012). Predictor Variables for Marathon Race Time in Recreational Female Runners. Asian J. Sports Med..

[11] Vernillo G., Schena F., Galvani C., Maggioni M.A. (2013). A thropometric characteristics of top class Kenyan marathon runners. J. Sports Med. Physic. Fit..

[12] Joyner M.J., Coyle E.F. (2008). Endurance exercise performance: The physiology of champions. J. Physiol..

[13] Jones A.M. (2006). The Physiology of the World Record Holder for the Women’s Marathon. Int. J. Sports Sci. Coach..

[14] Chmura J., Chmura P., Konefat M., Batra A., Mroczek D., Kosowski M., Mlynarska K., Andrzejewski D., Rokita A., Ponikowski P. (2020). The effects of a marathon effort on psychomotor performance ad catecholamine concentration in runners over 50 years of age. Appl. Sci..

[15] Hernando C., Hernando C., Collado E.J., Panizo N., Martinez-Navarro I., Hernando B. (2018). Establishing cut-points for physical activity classification using triaxial accelerometer in middle-aged recreational marathoners. PLoS ONE.

[16] Hernando C., Hernando C., Martinez-Navaro I., Collado-Boira E., Panizo N., Hrenando B., Hernando B. (2020). Estimation of energy consumed by midlle aged recreational marathoners during a marathon using accelerometry-based devices. Sci. Rep..

[17] Beneke R., Leithäuser R.M., Ochentel O. (2011). Blood Lactate Diagnostics in Exercise Testing and Training. Int. J. Sports Physiol. Perform..

[18] Sjödin B., Svedenhag J. (1985). Applied Physiology of Marathon Running. Sports Med..

[19] Christensen C.L., Ruhling R.O. (1983). Physical characteristics of novice and experienced women marathon runners. Br. J. Sports Med..

[20] Haney T.A., Mercer J. (2011). A Description of Variability of Pacing in Marathon Distance Running. Int. J. Exerc. Sci..

[21] www.athensauthenticmarathon.gr.

[22] www.plotaroute.com/routeprofile/20092.

[23] Cooke C., Eston R., Reilly T. (2009). Maximal oxygen uptake, economy and efficiency. Kinanthropometry and Exercise Physiology Laboratory Manual.

[24] Péronnet F., Massicotte D. (1991). Table of nonprotein respiratory quotient: An update. Can. J. Sport Sci..

[25] Jeukendrup A.E., Wallis G.A. (2005). Measurement of Substrate Oxidation during Exercise by Means of Gas Exchange Measurements. Int. J. Sports Med..

[26] Joyner M.J., Hunter S.K., Lucia A., Jones A.M. (2020). Last Word on Viewpoint: Physiology and fast marathons. J. Appl. Physiol..

[27] Joyner M.J. (1991). Modeling: Optimal marathon performance on the basis of physiological factors. J. Appl. Physiol..

[28] Lucia A., Esteve J., Olivan J., Gallego-Gomez F. (2006). Physiological characteristics of the best Eritrean runners—Exceptional economy. Appl. Physiol. Nutr. Met..

[29] Foster C., Lucia A. (2007). Running economy: The forgotten factor in elite performance. Sports Med..

[30] Saunders P., Pyne D., Telford R., Hawley J. (2004). Factors affecting running economy in trained distance runners. Sports Med..

[31] Barnes K., Kilding A. (2015). Running economy: Measurement, norms, and determining factors. Sports Med. Open.

[32] Hoogkamer W., Kram R., Arellano C.J. (2017). How Biomechanical Improvements in Running Economy Could Break the 2-h Marathon Barrier. Sports Med..

[33] Lee E., Snyder E., Lundstrom C. (2020). Effects of marathon training in maximal aerobic capacity and running economy in experienced marathon runners. J. Hum. Sport Exerc..

[34] Barnes K.R., Hopkins W.G., McGuigan M.R., Northuis M.E., Kilding A.E. (2013). Effects of Resistance Training on Running Economy and Cross-country Performance. Med. Sci. Sports Exerc..

[35] Kolbe T., Dennis S., Selley E., Noakes T., Lambert M. (1995). The relationship between critical power and running performance. J. Sports Sci..

[36] Noakes T.D., Myburgh K.H., Schall R. (1990). Peak treadmill running velocity during theVO2max test predicts running performance. J. Sports Sci..

[37] Tokmakidis S.P., Léger L.A., Pilianidis T.C. (1998). Failure to obtain a unique threshold on the blood lactate concentration curve during exercise. Eur. J. Appl. Physiol. Occup. Physiol..

[38] Davies C.T.M., Thompson M.W. (1979). Aerobic performance of female marathon and male ultramarathon athletes. Eur. J. Appl. Physiol. Occup. Physiol..

[39] Maughan R.J., Leiper J.B. (1983). Aerobic capacity and fractional utilization of aerobic capacity in elite and non-elite male and female marathon runners. Eur. J. Appl. Physiol. Occup. Physiol..

